# Investigation of Risk Factors Predicting Cataract Surgery Complications in Patients with Pseudoexfoliation Syndrome: A Systematic Review

**DOI:** 10.3390/jcm13061824

**Published:** 2024-03-21

**Authors:** Laura Denisa Preoteasa, George Baltă, Florian N. Baltă

**Affiliations:** 1 Department of Ophthalmology, Carol Davila University of Medicine and Pharmacy, 020021 Bucharest, Romania; 2 Department of Ophthalmology, Clinical Emergency Eye Hospital, 010464 Bucharest, Romania; 3 Onioptic Hospital, 200136 Craiova, Romania

**Keywords:** pseudoexfoliation syndrome, cataract surgery complications, prognosis, risk factors

## Abstract

**(1) Background:** The present review aims to identify risk factors with predictive value for differentiating between pseudoexfoliation patients at risk of developing intra- or postoperative complications and those without operative risk during cataract surgery. **(2) Methods:** The review protocol was registered at PROSPERO, registration no. CRD42023417721. The following databases were searched for studies between 2000 and 2023: PubMed/Medline, Scopus, Springer, Science Direct, Web of Science, Cochrane Database of Systematic Reviews, TRIP database, LILACS, Clinical Trials, and reference lists of articles. We included analytical studies of any design examining cataract surgery complications in pseudoexfoliation patients across two population groups, one who underwent uneventful cataract surgery and the other who experienced intra- or postoperative complications. The paper will follow PRISMA 2020 criteria for reporting. Effect measure was assessed using odds ratios (ORs) and corresponding 95% confidence interval (CI) for qualitative variables and means with their respective standard deviation (SD) for quantitative variables. The risk of bias was assessed using the method presented in the Cochrane Handbook for Systematic Reviews. The GRADE scale was used for quality of evidence and certainty. **(3) Results:** The initial search of published and gray literature databases retrieved 1435 articles, six of which were included in this report. A total of 156 intra- or postoperative incidents were reported in 999 eyes with pseudoexfoliation. The identified predictive factors were a shallow anterior chamber, cataract grade, neutrophil-to-lymphocyte ratio, preoperative intraocular pressure, and symmetry of the exfoliation material. Limitations include heterogeneity of data and limited number of studies identified in our search. **(4) Conclusions:** These findings suggest the potential to refine risk stratification protocols in clinical settings and assist surgeons in personalized decision-making among individuals with pseudoexfoliation syndrome.

## 1. Introduction

Pseudoexfoliation syndrome (PXF) represents a medical condition with a complex pathology and an etiology that has not been fully elucidated. This syndrome encompasses a spectrum of ocular, surgical, and systemic complications, making it a multifactorial clinical entity.

It is found in approximately 10–20% of the general population older than 50 years [1]. The global incidence of this disease varies widely, with a prevalence ranging between 1.5% and 40.9% worldwide [2,3]. Certain geographic regions and ethnic groups are predisposed to the condition [1]. PXF incidence varies between 3.6% and 34.2% in Europe, between 1.5% and 22.1% in Asia, and between 1.5% and 40% in African countries, suggesting a general lack of consensus in these epidemiological studies [2,3]. The highest incidence has been recorded in Scandinavia, where approximately half of open-angle glaucoma cases are attributed to pseudoexfoliation syndrome [4].

Etiopathogenesis involves the appearance of exfoliation material adhering to the anterior capsule of the crystalline lens, the posterior epithelium of the iris and ciliary body, the zonule of Zinn, and the anterior surface of the vitreous [5]. Electron microscopy revealed characteristic fibrils within epithelial cells embedded in an amorphous matrix [6]. The fibrillar material, possibly the proteoglycan/glycoprotein complex, has an undetermined composition [6]. Ischemia, hypoxia, oxidative stress, and chronic inflammation are additional pathogenic factors [5,7,8]. However, whether the accumulation of pseudoexfoliative material results from excessive production or inefficient degradation has not been determined.

The deposition of fibrillar material leads to zonular instability, causing dislocation or subluxation of the natural crystalline lens [9]. It also contributes to pseudoexfoliative glaucoma when deposited in the trabecular meshwork [9]. Systemic complications associated with PXF syndrome include changes in collagen and elastin within vessel walls (hypertension, myocardial infarction, stroke, Alzheimer’s disease, diabetes) and extraocular connective tissue (benign prostatic hyperplasia, chronic kidney disease, chronic obstructive pulmonary disease, and inner ear dysfunctions) [2,10,11,12,13,14].

Over the past decade, a growing number of studies have focused on differences between populations with and without pseudoexfoliation syndrome [15,16,17]. However, not all PXF patients develop surgical complications, and additional data are needed to identify risk factors that increase the rate of intra- or postoperative complications in patients with pseudoexfoliation syndrome. To the best of our knowledge, no systematic reviews have investigated predictive factors for complications related to pseudoexfoliation in cataract surgery.

Cataract surgery complications not only have immediate consequences for patients in terms of suboptimal visual outcomes and visual disturbances, but also contribute to the overall burden on the healthcare system, including increased demand for ophthalmic services, surgical capacity, and postoperative care. Patients experiencing complications may require more frequent follow-up visits and sometimes even additional surgeries, leading to increased healthcare costs and allocation of further resources. The economic burden associated with complications extends beyond direct healthcare costs and can include indirect costs related to productivity loss, caregiver burden, and disability.

We performed a systematic review that aimed to identify risk factors linked to PXF syndrome that may contribute to intraoperative or postoperative complications. We also aimed to determine the factors with predictive value for differentiating between pseudoexfoliation patients without operative risk and those at risk of developing complications. This approach can improve surgical decision-making, enhance patient counseling, and better allocate patients to surgeons based on their skills. Providing patients with information about their specific risk profile allows for better-informed consent discussions, manages patient expectations, and potentially improves patient satisfaction.

## 2. Materials and Methods

The review protocol was registered at the PROSPERO International Prospective Register of Systematic Reviews (https://www.crd.york.ac.uk/prospero, accessed on 27 April 2023), registration No. CRD42023417721. The paper will follow the PRISMA 2020 criteria for reporting [18].

The following databases were searched for studies published between 2000 and 2023: PubMed/Medline, Scopus, Springer, Science Direct, Web of Science, Cochrane Database of Systematic Reviews, TRIP database, LILACS, and Clinical Trials. The reference lists of reviews and clinical trials were scanned for relevant citations that might have been missed. The search strategy included the following terms (and synonyms): cataract surgery; cataract extraction; complication; incidence; risk factor; prognostic factor; prognosis; pseudoexfoliation; pex; pseudoexfoliation syndrome; pseudoexfoliative material. Articles were found using both open-text fields and medical search headings.

Regarding eligibility, analytical studies of any design with human participants were selected. The study included people older than 40 years who were clinically diagnosed with pseudoexfoliation syndrome and who underwent standard cataract surgery through phacoemulsification. Only studies that provided a statistical association between exposure and clinical course were included. The exclusion criteria for patients were as follows: (1) under 40 years old and older than 40 years old with increased surgical risk (history of trauma, corneal opacities, Fuchs endothelial dystrophy, aphakia, previous vitreoretinal surgery, other types of cataracts, unable to undergo standard cataract surgery), (2) underwent surgery by trainees or residents, (3) data not reliably extracted, duplicate, or overlapping data, (4) were nonhuman studies or in vitro studies, (5) were abstracts-only papers, such as preceding papers, conference, editorial, and author response theses and books, and (6) were case reports, case series, and systematic review studies. To reduce potential bias, no restrictions were placed on sex, country, ethnicity, publication language, or sample size. Where possible, the authors of the articles were contacted to request missing data [19].

Two independent reviewers (L.D.P and G.B.) tabulated and extracted the data from the studies. In case of disagreement, issues were discussed with the participation of a third-party reviewer (F.B.) as needed. A structured Microsoft Excel form was used to extract the relevant data from each of the articles. The year of publication, study design, duration of follow-up, number of individuals in each group who completed follow-up (sample size), mean age of participants with their standard deviation and 95% CI, number of cases and controls, definition of control sample, exposure as a specific risk factor with their OR and corresponding 95% confidence interval (CI) for qualitative variables and means with their respective standard deviation (SD) for quantitative variables, intra- or postoperative complications as outcome, adjustment variables, and quality assessment tool (QA) were all recorded characteristics of these studies.

**Figure 1:**
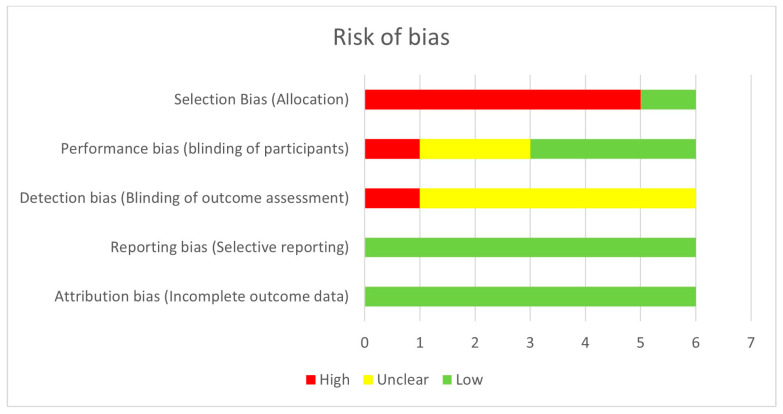
Assessment of risk of bias using the method of the Cochrane Handbook for Systematic Reviews.

The method presented in the Cochrane Handbook for Systematic Reviews was used to assess the risk of bias [20]. L.D.P. and G.B. independently evaluated each included study to identify potential sources of bias. The authors categorized bias as low, high, or unclear risk. Discrepancies were resolved through discussion, and any unresolved disagreements were resolved by a third author (F.B.). The potential risks of bias considered included selection bias (sequence generation, allocation concealment), performance bias (blinding of participants and study personnel), detection bias (blinding of outcome assessment), attribution bias (incomplete outcome data), and reporting bias (selective outcome reporting)—Figure 1, Table 1. To address missing or unclear data, we contacted the study authors for additional information [19]. The quality of evidence and certainty was assessed using the GRADE scale [21]. The GRADE scale assesses risk of bias within a study (methodological quality), directness of evidence, heterogeneity, precision of effect estimates, and risk of publication bias. High quality studies start as prospective analytical studies, while retrospective studies are upgraded if they produce large effects with no obvious bias. Conversely, prospective studies are downgraded in case of methodology heterogeneity, small sample size (*n* < 100), and risk of publication bias.

For each prognostic factor, we will present the measure of association with the corresponding adverse effect. ORs with corresponding 95% CIs were used to quantify qualitative variables, while means with SDs were used to represent quantitative variables. The initial analysis involved pooling outcomes from all studies (cohorts and case-controls), assuming that ORs are good estimates of relative risk. Study-specific ORs were combined or separated using inverse-variance fixed-effects or random-effects models, selecting the most conservative model. Heterogeneity was assessed using the I2 statistic [27], and an I2 of more than 50% indicated high heterogeneity [28].

## 3. Results

The review included studies of any design examining cataract surgery complications in pseudoexfoliation patients in at least two population groups, one who underwent uneventful cataract surgery and the other who developed intra- or postoperative complications. We searched for prognostic factors for a high risk of complications during cataract surgery that demonstrated a statistical association with intra- or postoperative cataract surgery complications.

**Figure 2:**
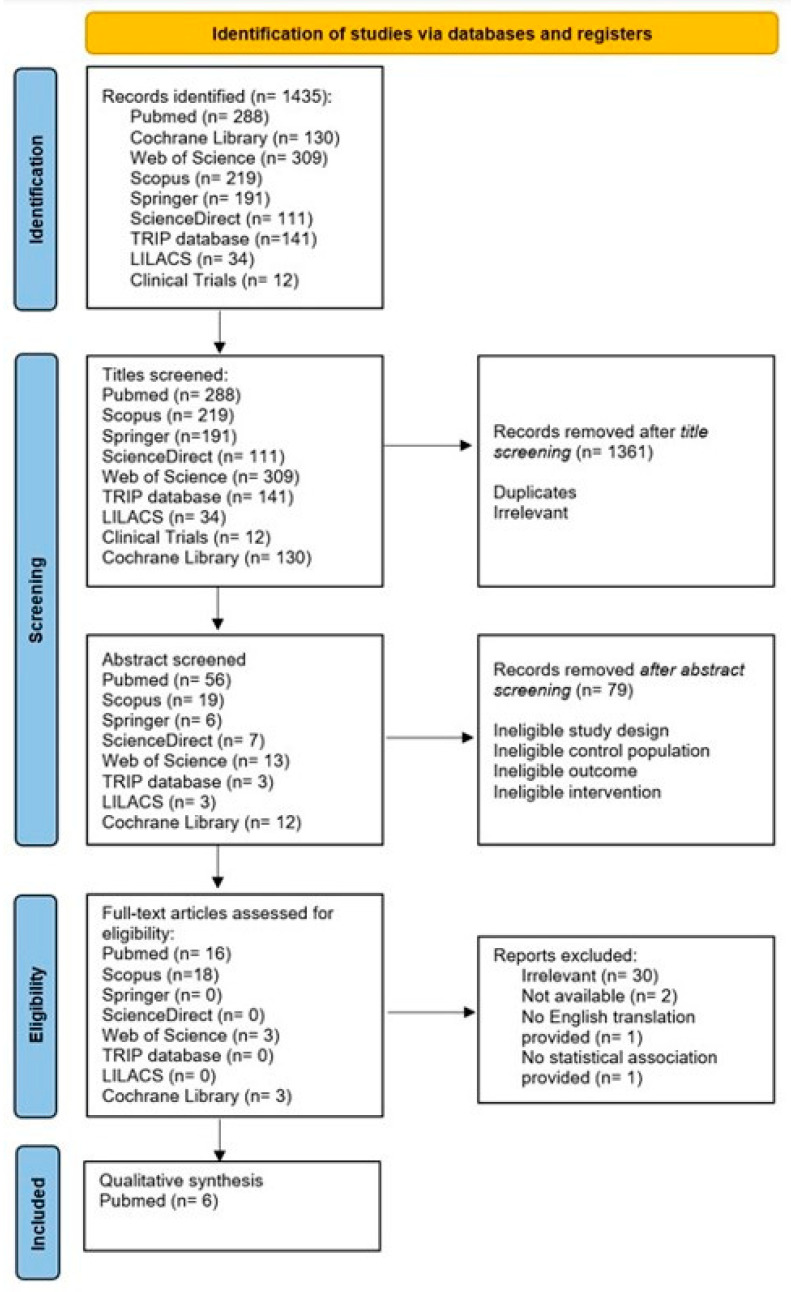
PRISMA flow chart of the process selecting the of reviewed articles.

The initial search of published and gray literature databases retrieved 1435 articles (Figure 2). After screening the abstracts, 40 articles were selected for full-text review. Out of 40 articles, 30 were considered irrelevant because of either an ineligible study design, a control population, the type of outcome reported, or the type of intervention; two articles were not provided with the full text, and one could only be found in the Russian language, even though the authors were contacted to offer an English translation of their paper. One immunohistochemical study investigated AGE carboxymethyl lysine (CML), yet it was removed because it did not show a statistical connection between clinical course and the immunohistochemical reaction of the PXF fibrils. The study concluded that CML is not a predictive factor for cataract surgery results [29]. Our analysis therefore included six articles (Table 2) [19,22,23,24,25,26]. There were two case-control studies [24,26] and four cohort studies [19,22,23,25]. Two studies were conducted in Europe [22,26] and four were conducted in Asia [19,23,24,25].

We included a total of 156 intra- or postoperative incidents reported in 999 eyes diagnosed with PXF. The mean age of our review was 74.77 years old ± 7.76 years. There was no significant difference in the mean age between studies. The male-to-female ratio was 373/417. The study details can be found in Table 2.

Two main groups of complications were evaluated: (1) intraoperative complications, defined as zonular dialysis and/or vitreous loss, zonular defects, posterior capsular rupture, and surprise phacodonesis; and (2) postoperative complications defined as corneal edema, wound burn, endothelial cell loss at 3 months, intraocular lens (IOL) position alterations, and capsular phimosis. We grouped “corneal edema”, “wound burn”, and “endothelial cell loss” as “endothelial cell decompensation” to avoid confusion. Three studies reported intraoperative complications [22,24,25], two studies reported postoperative complications [19,23], and one study reported both types of complications [26] (Table 3).

The available data on outcomes are rather heterogeneous. Two studies considered the shallow anterior chamber (ACD) a risk factor [19,22], two studies proved that late-stage cataracts according to the Emery–Little lens opacity classification system may predict postoperative complications [19,23], one study focused on systemic inflammatory markers (NLR—neutrophil-to-lymphocyte ratio) to predict surgical events [24], one study proved that an intraocular pressure (IOP) above 23 mmHg can be a risk factor [25], and one study proved that PXF symmetry can influence surgical outcome [26]. (Table 3).

An inverse variance-weighted random-effects meta-analysis of the adverse effects on the outcomes was performed. We obtained a Q statistic = 93.48 and an I2 = 93.58, indicating high heterogeneity.

## 4. Discussion

Despite conducting a systematic review of the literature on complications in PXF, we found that only six relatively small studies contributed to the understanding of clinical decision-making. Most published studies involving pseudoexfoliation patients used the normal population without PXF as the control group and compared risk factors between PXF and non-PXF patients. To our knowledge, this is the first systematic literature review investigating exposure in patients diagnosed with pseudoexfoliation syndrome, which can lead to intra- or postoperative complications during cataract surgery.

The mean sample size of our review was 166.5 eyes, which is rather low, with fewer than 200 patients analyzed in most studies. There was no significant difference in the mean age of each study, which express similar populations. The mean age of our review was 74.77 years ± 7.76 years. The mean follow-up was 6.66 years. Postoperative complications can be categorized as “immediate”, occurring in the first days after surgery, and “delayed”, typically manifesting more than 5 years after surgery [30]. While immediate intra- and postoperative adverse effects are reliable, long-term complications, such as IOL dislocation or capsular phimosis, were limited in our assessment.

The literature recognizes pseudoexfoliation syndrome as a risk factor in cataract surgery. A meta-analysis comprising 22 studies revealed a twofold greater risk of intraoperative complications, including posterior capsule rupture or zonular dialysis, during phacoemulsification in patients with pseudoexfoliation [31]. Postoperative complications such as corneal edema, intraocular hypertension, and postoperative uveitis also demonstrate statistically significant differences between PXF patients and non-PXF patients [32]. However, the improvement in postoperative visual acuity was similar for patients with and without preoperatively identified pseudoexfoliative material [32].

Contradicting the idea that pseudoexfoliation syndrome poses additional risks, V. Sarda et al. found no statistically significant differences in operative incidents or postoperative visual acuity compared to a control group [14]. The obtained data were consistent with those published by Hisham Jammal, who did not identify any additional risk factors for posterior capsule rupture, vitreous loss, or zonular dehiscence in patients with pseudoexfoliation syndrome [33]. Jammal developed a predictive model (χ^2^ (5) = 21.54, *p* < 0.01) that included age, laterality, diabetes status, and lens instability as predictive factors for the development of complications in cataract surgery [33]. However, the only variables that significantly predicted surgical complications were age and sex, ruling out pseudoexfoliation syndrome as a risk factor [33]. The authors highlighted a decreasing trend in complication incidence over recent years, attributing this to advancements in surgical equipment and techniques.

Our review included two studies that considered shallow ACD as a risk factor [19,22], two studies that evaluated cataract grade according to the Emery–Little lens classification system as an outcome [19,23], one that analyzed the level of neutrophils and lymphocytes [24], one based on high IOP [25], and one examining the symmetry of pseudoexfoliative material for predicting clinical course [26].

Küchle established a cutoff value of 2.5 mm for anterior chamber depth (ACD) [22]. Eyes with an anterior chamber depth of less than 2.5 mm had a 13.4% risk of intraoperative complications (vitreous loss or zonular dialysis), whereas eyes with an anterior chamber depth of 2.5 mm or more had a lower risk of 2.8% Similarly, Hasegawa performed a factor analytical study of multiple parameters. The study revealed that the endothelial cell density (ECD) in patients with PXF is influenced after small-incision cataract surgery [19]. The analysis resulted in a notable correlation (R^2^ = 0.12, *p* = 0.006). The final linear regression model included significant parameters, namely, cataract grade (*p* = 0.019) and preoperative ACD (*p* = 0.023). The ECD was reduced by 3.45% with a 1 mm decrease in the preoperative ACD [19]. However, morphological analysis, including the percentage of hexagonal cells and variation in the cell size of corneal endothelial cells, was not performed, as these parameters may be more sensitive indicators. Another limitation of Hasegawa’s study was the lack of a control group. The contribution of ACD to endothelial cell damage is still controversial. Hasegawa concluded that the reduction in endothelial cells in eyes with pseudoexfoliation (PEX) is mainly due to surgical invasion itself, and it remains unclear whether the presence of PEX contributes additionally to the loss of endothelial cells [19].

Jiang divided 86 patients into three categories: early-stage (grade II lens opacities and mild PXF deposits), middle-stage (grade III nucleus opacity and middle PXF deposits), and late-stage (grade VI to V cataract and severe pseudoexfoliation deposits) and found a statistically significant difference in corneal edema and wound burn among the three groups (*p* < 0.05, χ^2^ test) [23]. However, a multivariate logistic regression analysis was not performed to determine whether cataract grade maintained its statistical value if other covariates, such as age or economic conditions, were considered. Another limitation is that there is no clear definition of “corneal edema” or “wound burn”, and another parameter, such as the endothelial cell count, may have been more reliable in the analysis. Likewise, Hasegawa reported a 1.39% reduction in ECD density with a step of Emery–Little classification [19]. However, he considered that hard nuclei require additional ultrasound power and time, resulting in a significant increase in endothelial cell damage, and the reduction in ECD resulting from cataracts should not be PEX-related [19].

The role of inflammatory markers in PXF pathology has been widely investigated [34,35,36]. Gökce identified an NLR cutoff value of 2.33 or higher in ROC analysis to predict surgical intraoperative complications (posterior capsular rupture, vitreous loss, and zonular dialysis) with a sensitivity of 87.5% and a specificity of 78.1% [24]. An elevated neutrophil-to-lymphocyte ratio was observed to signify increased inflammatory activity in various ocular diseases [37,38]. Even if Gökce’s study was retrospective and had a low number of events, it still provided statistically significant outcomes, as did previous research confirming the inflammatory nature of PXF syndrome [36,39].

Buhbut identified two factors predicting intraoperative phacodonesis in univariate analysis: IOP > 21 mmHg (OR: 18.5, 95% CI: 4.18–81.84, *p* < 0.001) and B-scan use for dense cataracts (OR: 4.53, 95% CI: 1.14–17.95, *p* = 0.031) [25]. However, after adjusting for confounders, only the baseline IOP remained statistically significant according to multivariate logistic regression (OR: 1.22, 95% CI: 1.04, 1.43, *p* = 0.014). Evaluating the likelihood of intraoperative phacodonesis through a high fit regression model using intraocular pressure yielded an area under the ROC curve (AUROC) of 0.749 (95% CI: 0.644, 0.822), accurately classifying 93.18% of cases [25].

Rodriguez-Una analyzed the importance of symmetry in pseudoexfoliation [26]. Surgical intraoperative (*p* = 0.04) and late postoperative (IOL position alterations, *p* = 0.03) complications were observed only in eyes in which PXF was present bilaterally [26].

The identified risk factors pose several implications for surgical planning and patient counseling. Cataract surgery in patients with shallow AC may need changes to the surgical technique such as using a maintainer instead of viscoelastic agents to deepen the chamber or perform central pars plana vitrectomy with triamcinolone staining in extreme cases. Advanced cataracts may require reduced ultrasound energy or even intracapsular lens extraction. Elevated NLR may indicate systemic inflammation and these patients may benefit from preoperative optimization of systemic health and targeted anti-inflammatory therapy. Surgeons may consider using an osmotic diuretic in patients with high preoperative IOP to avoid further complications. Symmetry of exfoliative material can indicate an advanced stage of PXF and surgeons should avoid aggressive manipulation near areas of zonular weakness or use capsular tension rings. Effective communication and patient education regarding the implications of these risk factors are crucial for informed decision-making and ensuring realistic expectations regarding visual outcomes.

It is also important to emphasize the limitations of the evidence included in our review. The studies showed several methodological flaws. Two of them had a small sample size [19,23], four reported a low number of events [22,23,25,26], and four were based on retrospective analysis [19,24,25,26]. This introduces bias risks linked to recording baseline data and potential selection bias, with large uncertainty in the results. Designing safety studies to capture these outcomes poses significant challenges due to the need for a large number of participants. The limited number of studies identified in our search can impact our findings and influence the overall quality of the review, leading to publication bias. The absence of clinical trials and an overrepresentation of cohort and case-control studies pose inherent bias risks. Nonrandomized studies require confounding factors to reduce the risk of bias, and our review included three studies that controlled for confounders using multivariate logistic regression [19,22,25].

Inconsistencies exist in the definitions of “pseudoexfoliation syndrome” and cataract diagnosis among studies. Surgeon blinding is unclear, and preoperative knowledge of the patient’s risk profile may influence the surgical approach. The variability in surgical technique, phacoemulsification technique, incision size, type of anesthesia, and use of iris hooks or capsular tension rings further complicates comparisons between studies. Unreported information adds to the challenge of effectively consolidating outcomes across studies.

A significant strength of our study lies in the comprehensive literature search, which identified all relevant reports transparently. We evaluated the methodological quality of the included studies using standard and detailed criteria. Considering these strengths, we are confident that the question under research is accurately represented by this study.

## 5. Conclusions

Despite the abundant literature regarding the increased risk of complications, there is a lack of data regarding their onset and predictive factors. The following review aims to identify additional risk factors in PXF patients, thereby serving as a starting point for developing predictive models related to patients with pseudoexfoliation. The identified predictive factors were a shallow anterior chamber, cataract grade, neutrophil-to-lymphocyte ratio, preoperative intraocular pressure, and symmetry of the exfoliation material. These findings suggest the potential to refine risk stratification protocols in clinical settings and assist surgeons in personalized decision-making among individuals with pseudoexfoliation syndrome. The limited data available require further research to enhance our understanding of its optimal management.

## Figures and Tables

**Table 1:** Risk of bias judgement approach using the method of the Cochrane Handbook for Systematic Reviews.

First Author	Criteria	Strength	Concern	Risk of Bias
Küchle [22]	Selection bias	The participants were selected based on specific criteria related to pseudoexfoliation syndrome and cataract surgery. Exclusion criteria were clearly defined. Prospective design.		Low
	Performance bias	Cataract surgeries were performed by a total of five experienced surgeons, which could reduce performance bias.	Variations in surgical techniques among surgeons could still introduce bias.	Low
	Detection bias	The assessment of intraoperative complications was performed immediately after surgery, potentially reducing detection bias.	The study does not mention surgeons’ blinding to patients’ characteristics or preoperative findings, which could introduce bias.	Unclear
	Attrition bias	There is no mention of loss to follow-up since the outcome assessment was performed immediately after surgery, reducing attrition bias.		Low
	Reporting bias	The article appears to report all the measured outcomes without selective reporting.		Low
	Other potential sources of bias	The study controlled for various factors such as age, gender, and preoperative conditions like glaucoma.	However, there could still be unmeasured confounders.	Low
Jiang [23]	Selection bias	Prospective design.	Unclear methodology—the method of patient selection and potential for selection bias are not clearly described in the study. The study was conducted in a specific population (Uygur patients from Kashi), which might limit its generalizability.	High
	Performance bias	The study has a consistent protocol for performing phacoemulsification across all participants by the same surgeon.	Potential variations in surgical technique or skill level might introduce bias. Unclear surgeon blinding.	Low
	Detection bias		No blinding of outcome assessors.	High
	Attrition bias	There is no indication in the article of any missing data or dropouts.	However, this information is not explicitly stated, so it is unclear if there was attrition bias.	Low
	Reporting bias	The article appears to report all the measured outcomes without selective reporting.		
	Other potential sources of bias		Small sample size and small number of events encountered. No definition of “moderate to severe corneal edema” and “wound burn” and how the attribution was made. No information for controlling confounders.	High
Hasegawa [19]	Selection bias	The study has inclusion and exclusion criteria clearly defined. Patients with specific criteria related to PXF and cataract surgery were included, while those with certain conditions were excluded.	Retrospective design.	High
	Performance bias	The surgical procedure appears to be standardized across patients, as it was performed by experienced surgeons using a consistent technique.	Details regarding blinding of patients, surgeons, or outcome assessors are not provided.	Unclear
	Detection bias	The method for measuring ECD is described, and it seems to be performed using appropriate equipment and techniques.	Blinding of the individuals assessing ECD are not provided. Lack of blinding could introduce bias if the assessors were aware of the patients’ group allocation (PXF vs. control) when measuring ECD.	Unclear
	Attrition bias	There is no indication in the article of any missing data or dropouts. The retrospective design lowers the likelihood of dropout.	However, this information is not explicitly stated, so it is unclear if there was attrition bias.	Low
	Reporting bias	The article appears to report all the measured outcomes without selective reporting.		Low
	Other potential sources of bias	The study controlled for various factors such as cataract grade, preoperative ACD, concomitance of DM.	The lack of a control group Retrospective design. Small sample size There could still be unmeasured confounders.	High
Gökce [24]	Selection bias	The study includes patients who underwent cataract surgery between January 2016 and January 2020 in their Department of Ophthalmology in Ankara City Hospital, indicating a specific patient population.	It is unclear how patients were selected for inclusion in the study. Retrospective design. The data were collected from medical records, which may introduce biases related to data completeness, accuracy, and consistency.	High
	Performance bias		It is unclear whether there was blinding of patients, surgeons, or outcome assessors.	Unclear
	Detection bias		Unclear blinding of outcome assessors.	Unclear
	Attrition bias	There is no indication in the article of any missing data or dropouts. The retrospective design lowers the likelihood of dropout.	However, this information is not explicitly stated, so it is unclear if there was attrition bias.	Low
	Reporting bias	The article appears to report all the measured outcomes without selective reporting.		Low
	Other potential sources of bias		There is no information for controlling confounders.	High
Buhbut [25]	Selection bias	The participants were selected based on specific criteria related to pseudoexfoliation syndrome and cataract surgery. Exclusion criteria were clearly defined.	Retrospective design. The data were collected from medical records, which may introduce biases related to data completeness, accuracy, and consistency.	High
	Performance bias		There is no description of surgical protocol. Details regarding blinding of patients, surgeons, or outcome assessors are not provided.	High
	Detection bias		It is unclear whether the assessment of “surprise phacodonesis” is standardized and blinded to preoperative parameters.	Unclear
	Attrition bias	There is no indication in the article of any missing data or dropouts. The retrospective design lowers the likelihood of dropout.	However, this information is not explicitly stated, so it is unclear if there was attrition bias.	Low
	Reporting bias	The article appears to report all the measured outcomes without selective reporting.		Low
	Other potential sources of bias		The author mentions variable controlling, but these variables are not explicitly described. Small number of events.	Unclear
Rodriguez-Una [26]	Selection bias	The participants were selected based on specific criteria related to pseudoexfoliation syndrome and cataract surgery.	No exclusion criteria. Retrospective design.	High
	Performance bias	Standardized surgical procedure	Unclear surgeon blinding.	Low
	Detection bias		Unclear blinding of outcome assessors.	Unclear
	Attrition bias	There is no indication in the article of any missing data or dropouts. The retrospective design lowers the likelihood of dropout.	However, this information is not explicitly stated, so it is unclear if there was attrition bias.	Low
	Reporting bias	The article appears to report all the measured outcomes without selective reporting.		Low
	Other potential sources of bias		Small number of events There is no information for controlling confounders.	

ACD = anterior chamber depth; ECD = endothelial cell decompensation; DM = diabetes mellitus; PXF = pseudoexfoliation syndrome.

**Table 2:** Study characteristics.

First Author	Year	Country	Study Design	Sample Size (Eyes)	PXF Eyes with Complications	PXF Eyes without Complications	GRADE	Risk of Bias
Küchle [22]	2000	Germany	CH prospective	174	12	162	High	Low
Jiang [23]	2015	China	CH Prospective	88	27	61	Low	High
Hasegawa [19]	2016	Japan	CH Retrospective	78	36	N/A	Low	High
Gökce [24]	2022	Turkey	CC Retrospective	210	32	178	Moderate	Unclear
Buhbut [25]	2023	Israel	CH Retrospective	127	10	117	Moderate	Unclear
Rodriguez-Una [26]	2023	Spain	CC Retrospective	322	39	228	Low	Low

CC = case-control, CH = cohort.

**Table 3:** Effect measure (EM) for various preoperative risk factors.

First Author	Event	No. of Events	Exposure	EM for Exposure	95% CI	Significance Level *p*
Küchle [22]	zonular dialysis and/or vitreous loss	12	ACD < 2.5 mm	Mean = 2.36 ± 0.44	1.81, 3.37	0.013
Jiang [23]	moderate to severe corneal edema	20	Late-stage cataract (Emery–Little lens opacities classification system)	OR = 4.16	1.45, 11.91	0.001
Jiang [23]	wound burn	7	Late-stage cataract (Emery–Little lens opacities classification system)	OR = 11.36	1.30, 99.07	0.026
Hasegawa [19]	ECD loss at 3 months > 2.6%	36	Shallow ACD	Mean = 3.06 ± 0.37	2.17, 4.08	0.023
Hasegawa [19]	ECD loss at 3 months > 2.6%	36	Cataract grade (Emery–Little lens opacities classification system)	Mean = 1.64 ± 0.97	-	0.019
Gökce [24]	posterior capsular rupture, vitreous loss, zonular dialysis	68	NLR > 2.33	Mean = 2.68 ± 0.73	2.58, 2.78	<0.001
Buhbut [25]	surprise phacodonesis *	10	preoperative IOP > 23 mmHg	OR = 1.22 (multivariate logistic regression)	1.04, 1.43	0.014
Rodriguez-Una [26]	IOL position alterations	10	Symmetric PEX	OR = 12.79	0.74, 220.36	0.03
Rodriguez-Una [26]	capsular phimosis	12	Symmetric PEX	OR = 6.67	0.85, 52.32	0.03
Rodriguez-Una [26]	intraoperative complications **	7	Symmetric PEX	OR = 9.00	0.51, 159.02	0.04

ACD = anterior chamber depth; ECD = endothelial cell decompensation; NLR = neutrophil-to-lymphocyte ratio; IOL = intraocular lens; IOP = intraocular pressure; PEX = pseudoexfoliation syndrome * Surprise phacodonesis is defined by the author as “unstable AC, eccentric nucleus displacement, difficult cortex removal with peripheral posterior capsular infoldings during aspiration” [25]; ** Intraoperative complications are described by the author as “capsular tear, zonular dehiscence, posterior capsular rupture with or without vitreous loss, hyphema, iris sphincter tear” [26].

## Data Availability

The datasets used and/or analyzed during the current study are available from the corresponding author upon reasonable request.
